# Rate-limiting hydrolysis in ribosomal release reactions revealed by ester activation

**DOI:** 10.1016/j.jbc.2022.102509

**Published:** 2022-09-20

**Authors:** Letian Bao, Victoriia V. Karpenko, Anthony C. Forster

**Affiliations:** Department of Cell and Molecular Biology, Uppsala University, Uppsala, Sweden

**Keywords:** protein synthesis termination, release factor, ribosome, fluorinated amino acid, ester hydrolysis, tRNA, FM, factor mix, GGQ, Gly-Gly-Gln, MeAla, methylalanine, PM, polymix, PTC, peptidyl transferase center, RC, release complex, RF, release factor, RM, ribosome mix, TFMeAla, trifluoromethylalanine

## Abstract

Translation terminates by releasing the polypeptide chain in one of two chemical reactions catalyzed by the ribosome. Release is also a target for engineering, as readthrough of a stop codon enables incorporation of unnatural amino acids and treatment of genetic diseases. Hydrolysis of the ester bond of peptidyl-tRNA requires conformational changes of both a class I release factor (RF) protein and the peptidyl transferase center of a large subunit rRNA. The rate-limiting step was proposed to be hydrolysis at physiological pH and an RF conformational change at higher pH, but evidence was indirect. Here, we tested this by activating the ester electrophile at the *Escherichia coli* ribosomal P site using a trifluorine-substituted amino acid. Quench-flow kinetics revealed that RF1-catalyzed release could be accelerated, but only at pH 6.2-7.7 and not higher pH. This provided direct evidence for rate-limiting hydrolysis at physiological or lower pH and a different rate limitation at higher pH. Additionally, we optimized RF-free release catalyzed by unacylated tRNA or the CCA trinucleotide (in 30% acetone). We determined that these two model release reactions, although very slow, were surprisingly accelerated by the trifluorine analog but to a different extent from each other and from RF-catalyzed release. Hence, hydrolysis was rate limiting in all three reactions. Furthermore, in 20% ethanol, we found that there was significant competition between fMet-ethyl ester formation and release in all three release reactions. We thus favor proposed mechanisms for translation termination that do not require a fully-negatively-charged OH^−^ nucleophile.

Stop codons within mRNAs are recognized during translation at the ribosomal A site by class I release factors (RFs), termed RF1 and RF2 in bacteria. RF1 binds UAA and UAG, whereas RF2 binds UAA and UGA. The bound RF then undergoes a conformational change that causes the peptidyl-tRNA ester electrophile at the P site to be attacked by a water or hydroxide nucleophile (1), and the polypeptide dissociates from the ribosome (Fig. 1*A*). After peptidyl release, the class II RF termed RF3 disassociates RF1 or RF2 from the ribosome and enables a recycling of protein synthesis by ribosome recycling factor (2).

**Figure 1:**
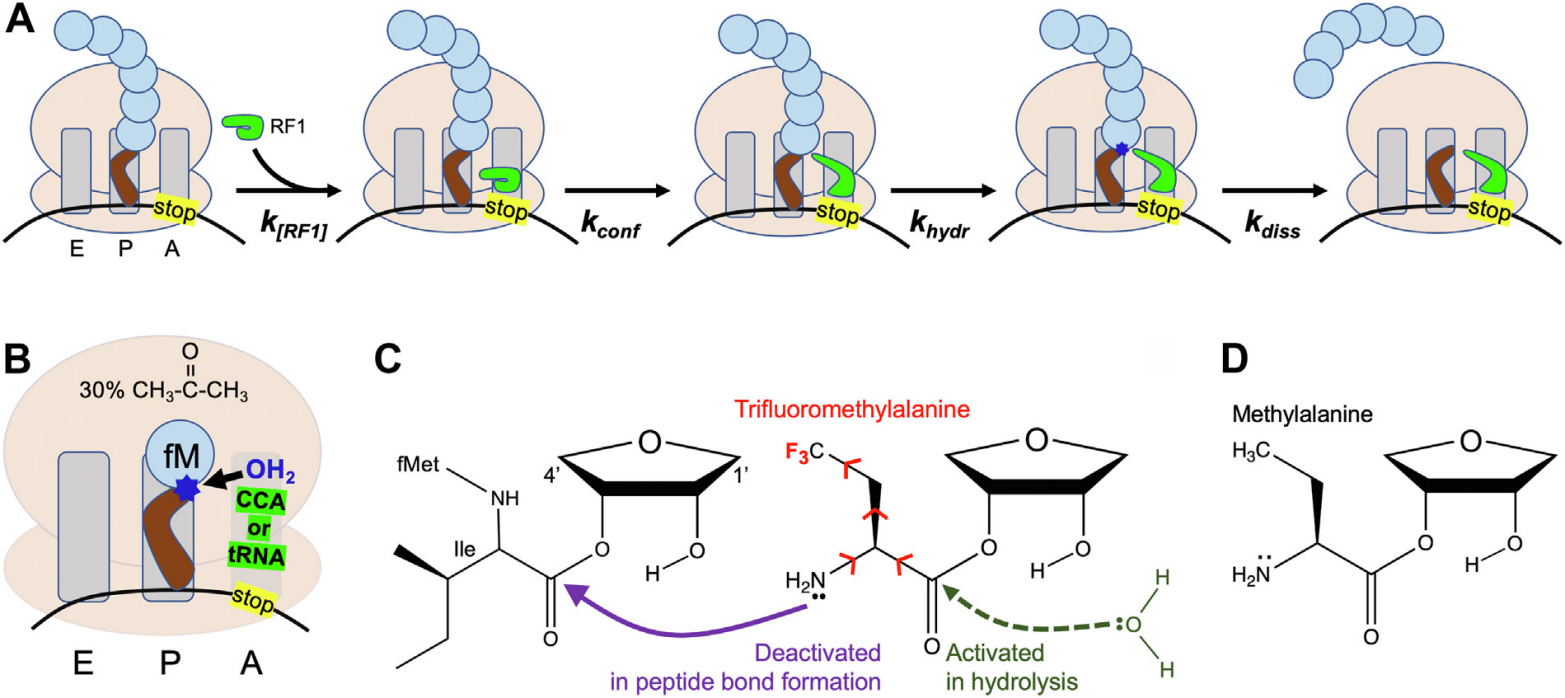
**Overview of ribosomal peptidyl-tRNA release reactions.***A*, release steps *in vivo*. The exit (E), peptidyl (P), and aminoacyl (A) sites of the ribosome are indicated, the tRNA is *brown*, and amino acids are *blue circles*. *k*_*conf*_, Rate of conformational change in RF1 to allow positioning of water for ester hydrolysis (*k*_*hydr*_) and subsequent dissociation of polypeptide (*k*_*diss*_). *B*, RF-free release reactions *in vitro* in 30% acetone. *C*, electron-withdrawing fluorine substitutions (*red*) of acylated TFMeAla amino acid that deactivate its amino group and activate the carbon of the ester group. In *C* and *D*, the adenine and tRNA bodies linked to the 1′ and 4′ positions, respectively, of the three ribose rings are not shown. *C*, depicts the second (and final) peptide bond formation (*purple*) in the preparation of the RC of Figure 2*A*. Subsequent attack (*dashed*) by the water nucleophile in the release reaction is shown in *green* and also in Fig. S1*B*. *D*, acylated MeAla unactivated control. MeAla, methylalanine; RC, release complex; RF, release factor; TFMeAla, trifluoromethylalanine.

Prokaryotic and eukaryotic class I RFs share the universally conserved motif Gly-Gly-Gln (GGQ), and mutation of each of these amino acids is lethal (3). *In vitro*, mutations in GGQ either decrease RF efficiency or induce nonspecific binding of nucleophiles (4) that correlate with lethality. Furthermore, the Gln side-chain amide is methylated in both prokaryotes and eukaryotes and the methylation is important for improving the accuracy of peptidyl release (5). Crystallography of free class I RF found that the distance between the GGQ and RF sequence that binds the stop codon was too little to span from the codon to the ribosomal peptidyl transferase center (PTC), but crystallography of the activated release complex (RC) showed that the RF had opened up to span the distance (1). Time-resolved cryo-EM further revealed that, within 24 ms of binding to the pretermination ribosomal complex at 25 °C, one-quarter of the RFs were in the compact conformation, whereas, within 60 s, virtually all RFs had opened (6). Although the water substrate has not been placed reliably, it was suggested that it hydrogen bonds to the methylated Gln to facilitate nucleophilic attack (7).

The RNA moieties of the PTC and peptidyl-tRNA substrate are also involved in release, which makes sense considering that ribosomal release and peptidyl transfer share the same ribosomal locations and similar mechanisms. The 2′OH of the peptidyl-tRNA 3′-terminal A76 was found to mediate substrate-assisted catalysis for both peptidyl transfer and release (8). Furthermore, release is abolished by mutation of the universally conserved A2602 of the PTC, although peptidyl transfer withstands this (9). Remarkably, efficient release was even possible *in vitro* if the RF was substituted with unacylated tRNA or CCA trinucleotide (10) (Fig. 1*B*), although 30% acetone was required. Crystallography showed that the CCA performed two functions: (i) binding the PTC to cause an activating conformational change nearly identical to that occurring for peptidyl transfer and (ii) positioning the water nucleophile to attack the ester (11).

Several catalytic mechanisms have been proposed for release (12, 13, 14) where the crucial A76 2′OH plays an identical role or a different role from that proposed in peptidyl transfer. Release or peptidyl transfer could either begin with deprotonation of the 2′OH or be concerted, and 4-, 6-, and 8-membered ring mechanisms are possible, depending on how many OH groups are directly involved. Both release and peptidyl transfer are accelerated by higher pH (12, 13, 15). The pH-sensitive group(s) in release is thought to be the nucleophilic water/OH^−^, nearby waters or the A76 2′OH, but other ribosomal or RF groups cannot be ruled out. Mindful that their results were consistent with titration of an unknown group with p*K*_a_ of 7.6, Indrisiunaite *et al.* (13) favored instead that their pH sensitivity below pH 7.6 reflected rate-limiting hydrolysis chemistry, whereas their lack of pH sensitivity above pH 7.6 reflected a rate-limiting conformational change of the RF. Another conformational change to consider is the activating one at the PTC (11) as this may also be pH insensitive and its rate is unknown. The rate of the actual release of a short peptide from the ribosome after hydrolysis (k_diss_ in Fig. 1*A*) was determined not to be rate limiting (see Table 2 of Ref. (13)) based on a comparison between quench-flow kinetics (which used radioactivity to measure up to the hydrolysis chemistry) and stopped flow kinetics (which used an N-terminal fluorophore to measure up to, and including, the dissociation of the free peptide from the ribosome). Thus, for clarity, we define release here as meaning all the steps in Figure 1*A* except k_diss_.

To summarize, the termination process in Figure 1*A* is likely to be rate limited by one of four steps: (i) binding of the RF to the ribosome, (ii) the conformational change of the RF from compact to open, (iii) the activating conformational change of the ribosomal PTC, or (iv) hydrolysis of the peptidyl-tRNA ester at the P site. *In vitro*, step (i) was generally not limiting because of using saturating RF, but (ii)–(iv) could not be distinguished conclusively. We reasoned that step (iv) could be monitored directly by making the unique ester in the RC more electrophilic. Previously, we found that a slow ribosomal peptide formation could be accelerated by incorporating three fluorines into the amino-acid side chain at the P site; hydrolysis assays off the ribosome found that this trifluoro substitution chemically activated the ester 11-fold (16). As ribosomal peptide bond formation is chemically related to peptidyl ester hydrolysis, we wondered if such an activated analog might also accelerate release (Fig. 1*C*, *red* and *green*; Fig. S1*B cartoon*).

Defining the rate-limiting step(s) in termination is important to understand experimental read out, *in vivo* accuracy of termination, and evolutionary bottlenecks for optimization. Also, this knowledge might aid applications with unnatural amino acids where their incorporation typically competes poorly with release (17) and aid therapies with drugs that compete with release (18).

## Results

### Chemical activation strategy to determine the rate-limiting step of ribosomal peptide release

**Figure 2:**
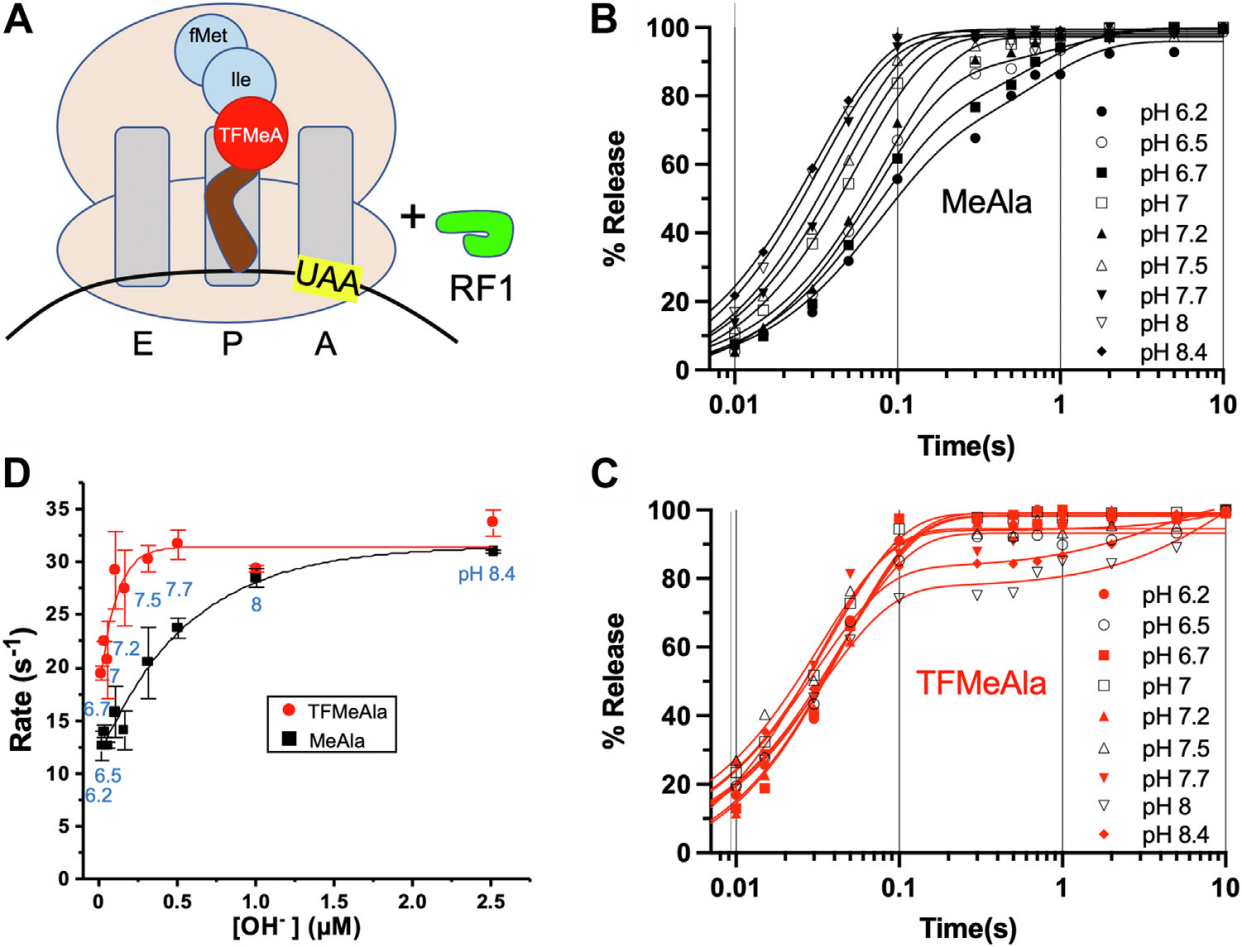
**RF1-catalyzed release with ribosomal tripeptide release complexes.***A*, kinetics reactions began by rapidly mixing RF1 with the activated-ester RC_UAA_ shown. For the unactivated control, MeAla was substituted into the third position (not shown). *B* and *C*, representative time courses at different pHs. *D*, peptide release rates calculated from the time courses are plotted against OH^−^ concentration. pHs are shown in *blue*. Spontaneous hydrolysis was subtracted in every experiment. Bars are SEs from at least two independent time courses. MeAla, methylalanine; RF, release factor; RC_UAA_, release complex with UAA codon at A site; TFMeAla, trifluoromethylalanine.

In contrast to many release assays that harbored only one amino acid at the P site and also our acceleration of peptide bond formation, here we mostly used purified RCs harboring tripeptides (Fig. 2*A*). This allowed for ready incorporation of radiolabel *via* the fMet and may be more physiological as a tripeptide is longer and showed 1.4- and 2.8-fold faster release than fMet by RF1 and RF2, respectively (13). In order to place our trifluoromethylalanine (TFMeAla) analog at the third position instead of the first, rather than irreversibly acetylating the amino group of TFMeAla (16), the pCpA-amino acid was acylated here with NVOC to allow photodeprotection (see the Experimental procedures section) and subsequent peptide bond formation. Two potential concerns with this tripeptide approach were (i) chemical activation of the analog’s ester is concomitant with partial *deactivation* of the amino nucleophile for prior peptide bond formation (Fig. 1*C*; *ignore the green*) and (ii) an RC with an activated ester (Fig. 2*A*) is less stable through synthesis, purification, and storage. In practice, however, using chemoenzymatically synthesized TFMeAla–tRNA^AlaB^_UGC_, we found that activated RCs could nevertheless be formed, purified, and stored with similar efficiency and residual activity to the control complex made with methylalanine (MeAla)–tRNA^AlaB^_UGC_ (Fig. 1*D*; see the Experimental procedures section).

### Ribosomal peptide release by RF1 at pH 7.5 is accelerated by ester activation

Armed with RCs to test for rate-limiting chemistry in release, rapid time points of the reaction were obtained by addition of saturating RF1 (5) in a quench-flow apparatus (Figs. 2*A* and S1). Interestingly, TFMeAla significantly accelerated the rate of release at pH 7.5 compared with MeAla (Fig. S1*C*, which is a subset of Fig. 2*D*). This was, to our knowledge, the first acceleration of release by substrate activation and the first direct evidence for rate-limiting hydrolysis chemistry. However, the activation was only 1.5-fold (*k*_obs_ = 30.4 ± 1.3 s^−1^ *versus k*_obs_ = 20.6 ± 3.4 s^−1^ in Table S1) compared with 11-fold chemical activation *per se* of very similar model compounds (off the ribosome) and 27-fold or no acceleration of slow and fast ribosomal peptide bond formations, respectively (16). A possible explanation for the smaller effect here is that accelerating hydrolysis unmasked another rate-limiting step. Alternatively, the fluorines may favor a suboptimal orientation at the P site for hydrolysis.

### Release by RF1 is accelerated by ester activation at lower, but not higher, pHs

Next, we varied the pH because the innate rate of alkaline hydrolysis of the ester should increase linearly with OH^−^ concentration, notwithstanding any potential effect of pH on another step. Indrisiunaite *et al.* (13) found that release increased linearly with OH^−^ concentration at lower pHs but saturated at higher pHs, hypothesizing that rate limitation switched at higher pH from hydrolysis to a pH-independent conformational change of RF1, thereby masking ever-faster innate hydrolysis on the ribosome. Our pH sensitivity of release rates with the control tripeptide containing MeAla was also hyperbolic, not linear, saturating at higher pHs (Fig. 2, *B* and *D*, *black*). It looked similar to that of Indrisiunaite *et al.* (13), except that our pH dependence was less marked at acidic pHs. A possible explanation for the loss of linearity at our extremes of pH is due to partial denaturation of the RC and/or RF1. For TFMeAla (Fig. 2*C*), release was accelerated compared with MeAla by up to twofold at lower to mid pHs but had minimal effect at higher pHs (Fig. 2*D* and Table S1). Saturation occurred at lower pHs for TFMeAla than MeAla, as might be expected by the ester activation accelerating hydrolysis but not a conformational change, that is, the rate-limiting step changing at higher pH. Paradoxically, even when innate TFMeAla ester hydrolysis was slowed at pHs below 7.5, the measured accelerations were still much less than predicted from the 11-fold chemical activation (only 1.6–2.0 times, but up to 2.9 times if subtracting for the apparent rate of conformational change; Table S1).

### RF-free peptidyl release for mechanistic studies

Ribosomal release catalyzed *in vitro* by tRNA or CCA instead of an RF (10), although nonphysiological, offers a useful mechanistic tool because the potentially rate-limiting RF conformational change is circumvented. Like the “fragment reaction” (peptide bond formation between an fMet–tRNA fragment and puromycin catalyzed by the 50S ribosomal subunit), which is only efficient in 30% ethanol or methanol solvent, RF-free release also requires organic solvent for efficiency. However, RF-free release experiments typically employed 30% acetone solvent instead of alcohols because alcohols are too nucleophilic and favor fMet-ester products over the more-physiological, standard, hydrolysis product, fMet.

As all published RF-free release assays used only fMet–tRNA at the P site and kinetics studies were minimal, we first surveyed experimentally various conditions to optimize the reactions. Although the reason for the requirement of organic solvent is unknown, it is plausible that it aids P-site binding (as established in the fragment reaction by Schmeing *et al.* (19)). Given that we used ribosomal tripeptide RCs here, which should be more stable than ribosomal fMet–tRNA initiation complexes, we wondered if the solvent requirement could be overcome. However, our tripeptide RCs still exhibited strong dependence (20–120 times stimulation) by organic solvent for release catalyzed by either unmodified tRNA^Phe^ or CCA (Figs. S2 and S3). For CCA-catalyzed release, we used the same RCs as for RF-catalyzed release, but for tRNA^Phe^-catalyzed release, we changed the mRNA stop codon (UAA) into a cognate A-site codon (UUC). Unexpectedly, another property of the acetone in the tRNA-catalyzed reactions was that it obviated the assumed need (20) for a cognate codon in the A site (Fig. S4). However, recognition of the CCA portion of the tRNA was necessary as expected, as removal of 3′-terminal CA of tRNA essentially abolished release (Fig. S4).

**Figure 3:**
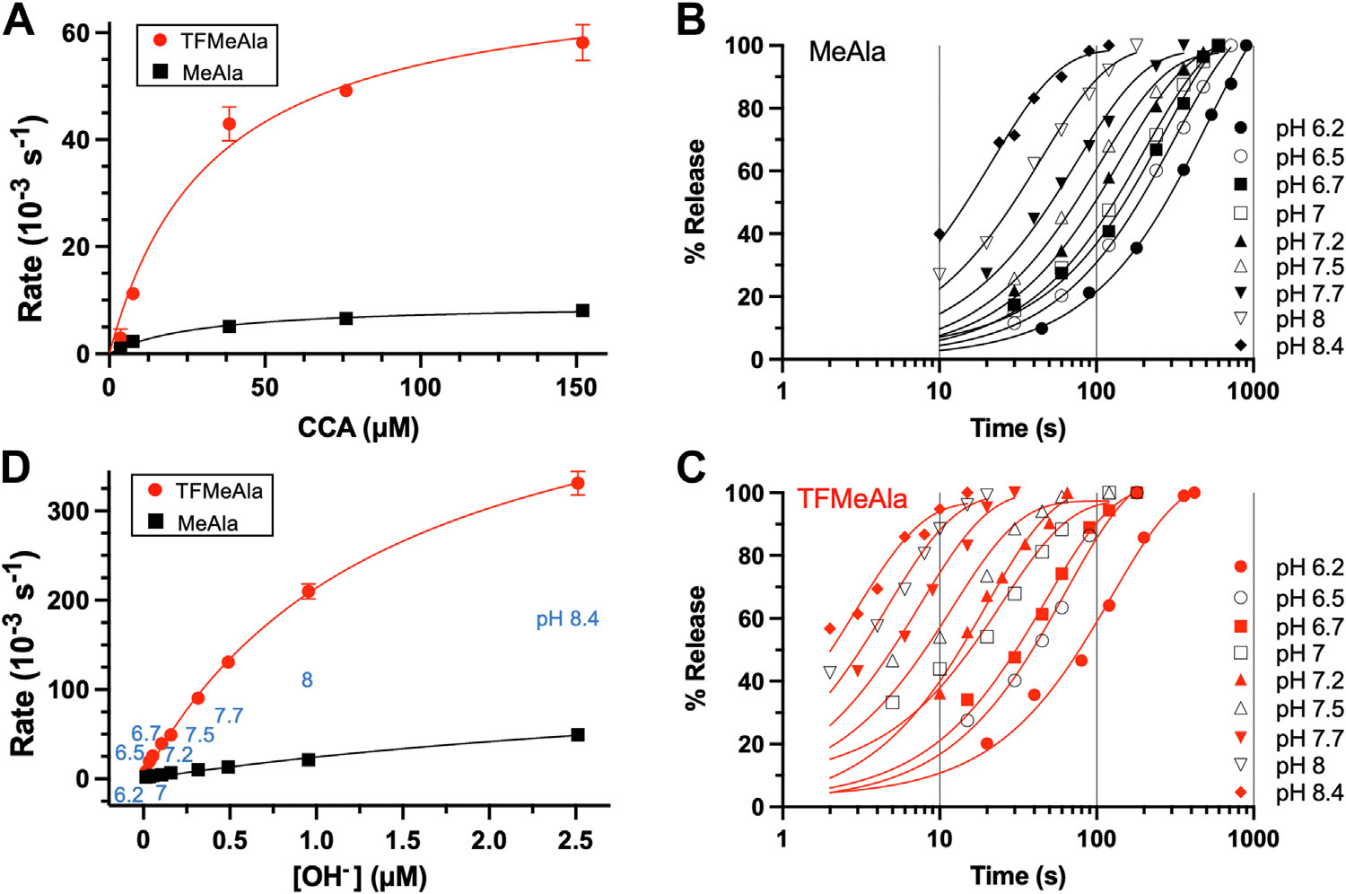
**CCA-catalyzed release with ribosomal tripeptide RCs in absence of RF1.***A*, concentration dependence of CCA. Time courses of CCA-catalyzed release of fMI-TFMeAla/MeAla tripeptides from RC_UAA_s (76 nM) at pH 7.2 in 30% acetone were measured, and the calculated rates were plotted. *B* and *C*, representative time courses of release catalyzed by CCA (76 μM) in 30% acetone at different pHs. *D*, release rates calculated from the time courses are plotted against OH^−^ concentration. SEs from at least two independent time courses. RC, release complex; RF, release factor; MeAla, methylalanine; TFMeAla, trifluoromethylalanine.

**Figure 4:**
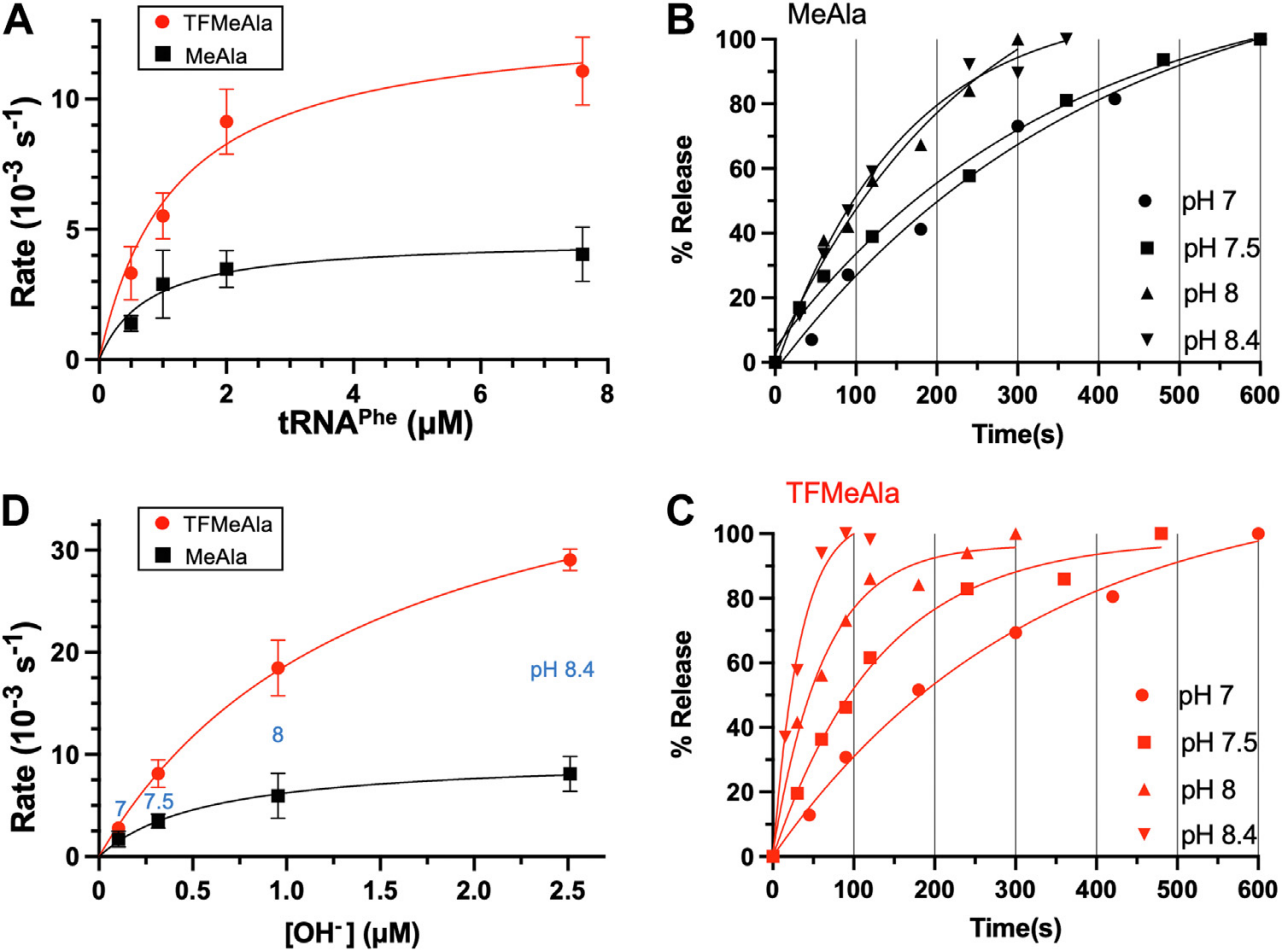
**tRNA**^**Phe**^**-catalyzed release with ribosomal tripeptide RCs in the absence of RF1.***A*, concentration dependence of tRNA^Phe^. Time courses of tRNA^Phe^-catalyzed release of fMI-TFMeAla/MeAla tripeptides from RC_UUC_s (76 nM with UUC Phe codon in the A site) at pH 7.5 in 30% acetone were measured, and the calculated rates were plotted. *B* and *C*, representative time courses of release catalyzed by tRNA^Phe^ (2 μM) at different pHs. *D*, release rates calculated from the time courses are plotted against OH^−^ concentration. SEs from at least two independent time courses. RC, release complex; RF, release factor; MeAla, methylalanine; TFMeAla, trifluoromethylalanine.

We titrated the concentrations of CCA and tRNA^Phe^ in the model release reactions with both unactivated and activated RCs (Figs. 3*A* and 4*A*). The *K*_*m*_ values with CCA for MeAla and TFMeAla (29.4 and 33.0 μM, respectively) were much higher than for tRNA^Phe^ (0.78 and 1.2 μM, respectively), as expected given that only the full tRNA could make most of the standard contacts at the ribosomal A site. Yet, despite the much lower binding affinity of CCA, use of saturating concentrations yielded much higher release rates with CCA than attainable with tRNA^Phe^ (for pH 7.5, see Figs. S5*D* and S6*D*). Nevertheless, these RF-free release reactions were more than three orders of magnitude slower than with RF (Table 1). Intriguingly, all three types of release reactions were accelerated by TFMeAla by different magnitudes (Table 1, *right*) and, in the case of CCA, acceleration was close to the previously measured 11-fold chemical activation. This indicated that hydrolysis, not a conformational change, was still the slowest step in these RF-free reactions, despite being more than three orders of magnitude slower than with RF.

**Table 1:** Summary of ribosomal release rates of tripeptides containing MeAla or TFMeAla catalyzed by saturating RF1, CCA, or tRNA^Phe^ at pH 7.5

Catalyst	*k*_obs_ MeAla (s^−1^)	*k*_obs_ TFMeAla (s^−1^)	Acceleration
RF1	20.6 ± 3.4	30.4 ± 1.3	1.5×
CCA	0.010 ± 0.0015	0.091 ± 0.0004	8.9×
tRNA^Phe^	0.0035 ± 0.0010	0.0081 ± 0.0023	2.3×

SEs are given. Acceleration factors are TFMeAla rates divided by MeAla rates.

### Release by tRNA^Phe^ or CCA increases almost linearly at low and physiological pHs and is accelerated by ester activation over the whole pH range

Given the acceleration of RF-free release reactions by TFMeAla at pH 7.5, we predicted that they would also be dependent on pH. Indeed, that was the case for CCA (Fig. 3, *B*–*D*) and tRNA^Phe^ (Fig. 4, *B*–*D*). These plots contrasted with that of RF1 where saturation of rates occurred at higher pHs (Fig. 2*D*). Also, unlike RF1, the accelerations by TFMeAla for both CCA and tRNA release reactions were substantial over the whole pH range (Tables S2 and S3). This implied rate-limiting hydrolysis over the whole pH range, consistent with the absence of the proposed rate-limiting pH-independent conformational change of RF1. A possible explanation for the moderate deviation from linearity at higher pH is due to partial denaturation of the RC.

### Competitive ester formation in release reactions in 20% ethanol catalyzed by RF1, CCA, or tRNA

**Figure 5:**
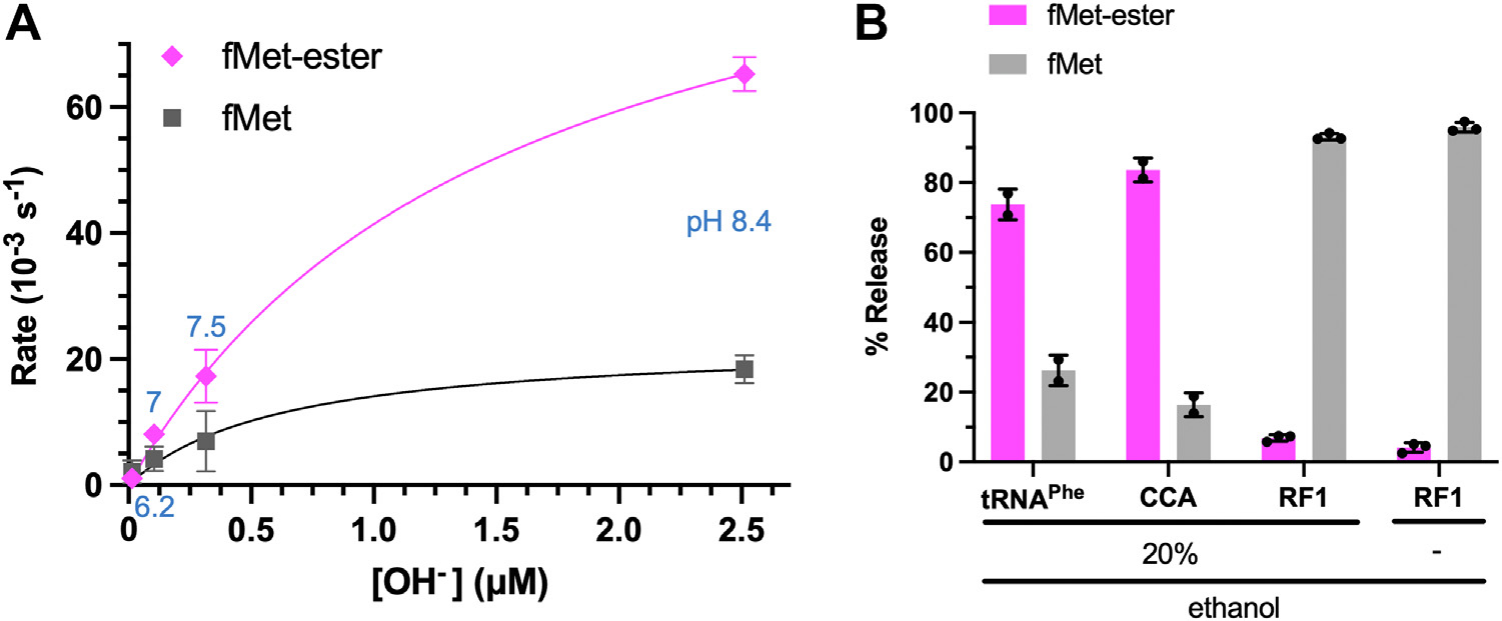
**Release of fMet *versus* fMet-ethyl ester formation in 20% ethanol catalyzed by RF1, CCA, or tRNA**^**Phe**^**.***A*, dependence on pH of tRNA^Phe^ (7.6 μM)-catalyzed formation of fMet-OEt *versus* fMet in 20% ethanol from RC (0.1 μM) harboring fMet–tRNA in the P site and a UUC Phe codon in the A site. Rates calculated from time courses are plotted against OH^−^ concentration. SEs from at least two independent time courses. *B*, product specificities in 20% ethanol at pH 7.5 catalyzed by RF1, CCA, or tRNA at 10, 480, and 600 s, respectively, reaction end times. tRNA^Phe^ (7.6 μM) was mixed with RC (0.1 μM) having a UUC Phe codon in the A site, whereas RF1 (4 μM) or CCA (76 μM) was mixed with RC (0.1 μM) having a UAA stop codon. SEs from at least two independent time courses. For RF1-catalyzed fMet-OEt formation at 20 *versus* 0% ethanol, the one-tailed *p* value = 0.023. RC, release complex; RF, release factor.

A fascinating feature of the RF1-free release reactions is that switching the solvent from aqueous acetone to aqueous ethanol switches them from ester hydrolysis to predominantly transesterification with ethanol (10). Given our finding that RF1-free release reactions were highly pH dependent, we wondered if ester formation would be also. The standard assays use an ^3^H-fMet–tRNA RC (Fig. 1*B*) in 20% ethanol followed by extraction of the fMet-OEt product into ethyl acetate (incompatible with tripeptide release assays). Figure 5*A* shows that these tRNA-catalyzed ester formations are also pH dependent in an almost linear manner without saturation at high pH, indicating rate-limiting deprotonation in ester formation.

The RF governed complete specificity *in vitro* for water/OH^−^ over ethanol as the nucleophile in 10% ethanol (4). But normally, at least 20% ethanol was used for ester formation, so we checked that condition. Surprisingly, RF1 enabled very rapid ester formation in 20% ethanol, although the Met release product was substantially more favored than in RF-free reactions (Fig. 5*B*). Thus, although we confirmed that RF governed the specificity of the nucleophile, we found that the specificity was not absolute.

## Discussion

We have opened a new window into rate-limiting steps in translation termination by chemically activating the scissile ester bond at the P site of ribosomal RCs. Whereas our analogous ribosomal approach only accelerated slow, not fast, peptide bond formations (16), here we accelerated a fast release reaction by substrate activation for the first time, to our knowledge. Although peptide bond formation and release are considered to be related reactions that both involve the PTC, the release is fourfold slower, which may be why we were more successful at accelerating release. The acceleration provides the first direct evidence that hydrolysis is the rate-limiting step in release at physiological pH, whereas the lack of acceleration at higher pH indicates that ever-faster hydrolysis becomes masked by a pH-insensitive slower step, presumably a conformational change of the RF, ribosome, or substrate.

Our results support the earlier combined proposal that hydrolysis is rate limiting at lower pH, whereas the known conformational change of the RF remains insensitive to pH and becomes limiting at higher pH (13). However, the complexity of the system and our data do not rule out other possibilities at higher pH. For example, crystallographic evidence showed that the PTC undergoes a conformational change for both peptide bond formation and release, and the rates of these steps are unknown. We have proposed rate-limiting “P-site accommodation” to explain why we only accelerated slow, not fast, peptide bond formations (16); such a model may also explain why our acceleration of release was a few-fold less than expected from the measured chemical activation in solution. Slow P-site accommodation in release might allow proofreading by protecting the RC from hydrolysis that is too rapid. Alternatively, the ester activation measured on the RC might be less than that of the innate chemical activation of the ester because of the unnatural amino acid causing mispositioning of the substrate on the ribosome, as apparently occurred in peptide bond formation assays (16, 21). Fluorine, although a good substitution for hydrogen, does differ in size and stereoelectronic properties in ways that could affect binding, even though the fluorines are in the amino acid side chain, which varies considerably between proteinogenic amino acids.

Old models of release were optimized and exploited as controls and to gain further mechanistic information. Release reactions substituting the RF by tRNA or CCA under saturating conditions showed acceleration by TFMeAla, an almost linear dependence on pH in the physiological range and did not saturate at higher pHs, implying rate-limiting hydrolysis. Although the rates were much slower than with RF, the plots were consistent with the idea that the hyperbolic curve observed with RF was not because of an intrinsic property of the ribosome, and that avoiding a rate-limiting RF conformational change could change the shape of the curve. A likely explanation for the slowing of the hydrolysis step is that neither a tRNA nor a CCA position the water substrate and surrounding water and RNA groups optimally for proton transfer. Results also demonstrated degrees of activation of the ester on the ribosome closer to that expected based on chemical activation off the ribosome, arguing that the ester of our activated RC was indeed highly activated in all assays. The different degrees of acceleration in the three types of release assays were puzzling (Table 1), with only the CCA assay being accelerated by the expected magnitude. Two ideas to explain this are that the fastest release (RF1 catalyzed) was at a similar rate to another very fast, almost rate-limiting step (like P-site accommodation), whereas the slowest release (tRNA catalyzed) was affected by tRNA also binding the E site (Fig. 1*B*) and adversely positioning the activated ester at the adjacent P site. However, the extents of acceleration for the RF1- and tRNA-catalyzed reactions were similar if the apparent rate of conformational change was subtracted from the RF1 rates (Table S1).

Finally, the known competition between ester hydrolysis and ester formation in tRNA-catalyzed release reactions in 20% ethanol (3.5 M ethanol, 44 M water) was found to be pH dependent, indicative of rate-limiting deprotonation. This ethanolic assay was extended to a more physiological condition by replacing the tRNA catalyst with RF. Interestingly, we found that there was still significant competition by ethanol with water for product formation, despite ethanol’s larger size and p*K*_a_. These results suggested to us that release is not facilitated by the RF selectively binding to a fully negatively charged OH^−^ nucleophile as postulated (13) because RF is unlikely to bind to ethanol/EtO^−^ as well as its natural substrate and the EtO^−^ nucleophile was at a lower concentration than OH^−^. Furthermore, OH^−^ and water are very difficult to distinguish by enzymes, notwithstanding that the concentration of OH^−^ at pH 7.5 = 0.3 μM, which is miniscule compared with water at 55 M in pure solution. Assuming that the natural substrate is water, the competitive ethanol reaction also suggests that one of water’s two polar OH bonds may not be directly involved. In summary, our data together with available evidence imply that release at physiological pH is rate limited predominantly by nucleophilic attack of the ester by water bound to the RF in a reaction involving rate-limiting deprotonation.

## Experimental procedures

### Materials

Pilot release kinetics were performed using 5,5,5-trifluoro-DL-leucine (Synquest Laboratories; 3x activated off ribosome). CCA trinucleotide was synthesized by Dharmacon, Inc. Tritium-labeled Met was purchased from PerkinElmer. All other chemicals and reagents were purchased from Sigma–Aldrich or Merck. *Escherichia coli*–methylated RF1 with a C-terminal His_6_ tag was co-overexpressed with untagged HemK methyltransferase and purified by a HisTrap High-Performance column (Cytiva) as previously described (13). Other translation factors, *E. coli* MRE600 70S ribosomes, and f[^3^H]Met-tRNA^fMet^ from overexpressed tRNA were prepared as described previously (16) (and references therein). All experiments were done at 37 °C in a Hepes–polymix (PM) buffer containing 5 mM Mg(OAc)_2_, 0.5 mM CaCl_2_, 95 mM KCl, 3 mM NH_4_Cl, 1 mM spermidine, 8 mM putrescine, 1 mM dithioerythritol, and 30 mM Hepes, in the absence or the presence of organic solvents. The pH of the PM buffer was adjusted with 0.5 M KOH or 1 M HCl.

### *N*-NVOC-MeAla-pCpA, *N*-NVOC-d,l-TFMeAla-pCpA, and chemoenzymatic aminoacylation of tRNA^AlaB^

*N*-NVOC-l-MeAla-pCpA and *N*-NVOC-d,l-TFMeAla-pCpA were prepared from l-MeAla and d,l-TFMeAla by substituting *N*-NVOC and pCpA (22) for the *N*-acetyl and pdCpA groups used in our prior derivatives (16) to now enable efficient ribosomal incorporation within the polypeptide chain. After chemoenzymatic ligation to *in vitro*-transcribed tRNA^AlaB^, the *N*-protected aminoacyl-tRNAs were purified by anion exchange Q Sepharose Fast Flow chromatography, and the NVOC protecting group was photolytically removed (22). Translationally active fractions of MeAla–tRNA^AlaB^ and TFMeAla–tRNA^AlaB^ were determined by yields of fMet–MeAla/TFMeAla dipeptide in 10 s and were similar (∼8–20%, varying from batch to batch) from the absorbances measured at 260 nm. Translation products were assumed to be from the l-amino acid isomers because d isomers react much more slowly at the ribosomal A and P sites (23).

### Preparation of mRNAs

Double-stranded DNA templates were prepared from synthetic oligodeoxyribonucleotides by primer extension, and *in vitro* transcription and mRNA purification were as described (16). The sequences were:

5′GGTACCGAAATTAATACGACTCACTATAGGGAATTCGGGCCCTTGTTAACAATT**AAGGAGG**TATTAA xxx xxx xxx xxx TTGCAGAAAAAAAAAAAAAAAAAAAAA3′ where the T7 promoter is underlined, the Shine–Dalgarno sequence is bold, and the underlined encoded Met-Ile-Ala-stop (ATG ATC GCA TAA), Met-Ile-Ala-Phe (ATG ATC GCA TTC), Met-Phe (ATG TTC), Met-stop (ATG TAA), or Met-Ala (ATG GCA).

### Preparation of RCs

A stalled ribosome RC carrying a peptidyl-tRNA with f[^3^H]Met-Ile-TFMeAla/MeAla tripeptide in the P site and a stop (UAA) or Phe (UUC) codon in the A site was prepared from a ribosome mix (RM) and a factor mix (FM) in Hepes–PM buffer (pH 7.5). RM contained 2 μM 70S ribosomes, 5 μM mRNA, 2.4 μM f[^3^H]Met-tRNA^fMet^, 2 μM IF1, 2 μM IF2, and 2 μM IF3. FM contained 10 μM EF-Tu, 5 μM EF-Ts, 5 μM EF-G, 100 μM tRNA bulk, 200 μM l-Ile, 2.4 μM MeAla/TFMeAla-tRNA^AlaB^, and 2 μM isoleucyl tRNA synthetase. Both RM and FM were supplied with 1 mM ATP, 1 mM GTP, 10 mM PEP, 0.02 g/l PK, 0.002 g/l MK, and an additional 2 mM Mg(OAc)_2_. RM and FM were preincubated separately at 37 °C for 20 min. The RC was formed by equally mixing RM and FM at 37 °C for 40 s and then chilled on ice. An additional 4 mM Mg(OAc)_2_ was added to stabilize the RC. Then RC was purified by ultracentrifugation on a 37.6% (m/v) sucrose cushion in a swing-out rotor (S55-S; Sorvall) at 258,000*g* for 4 h at 4 °C. The pellet containing RC was later dissolved in Hepes–PM buffer (pH 7.5).

### Quench-flow assay for RF1-catalyzed ribosomal peptide release

RCs harboring fMI-MeAla/TFMeAla_UAA_ prepared previously (0.2 μM) were mixed with an equal volume of saturating (5) methylated RF1 (6 μM) in a quench-flow apparatus (RQF-3; KinTeck Corp) at 37 °C and quenched by 17% formic acid at given time points. The quenched samples were cooled on ice, and supernatants containing released tripeptide were separated from pellets containing the tripeptidyl-tRNA by centrifugation at 20,000*g* for 30 min at 4 °C. Pellets were dissolved in 0.5 M KOH at 37 °C for 15 min. The radioactivity of dissolved pellets and supernatants were diluted in ProFlow G+ scintillation liquid for scintillation counting (Beckman Coulter LS 6500). The fraction of release was calculated as:

Release fraction = [^3^H] supernatant/([^3^H] supernatant + [^3^H] pellet)

Kinetic rates were estimated by double exponential decay function fitting

$$\text{y}=\text{A}1\text{∗exp}\left(-{\text{x/t}}_{1}\right)+\text{A}2\text{∗exp}\left(-{\text{x/t}}_{2}\right){+\text{y}}_{\text{0}}$$

where y is the release fraction; x is the time, A is the amplitude of the individual phases; t is the time constant; y_0_ is the offset, and 1/t is the rate constant (k). All release experiments were conducted at least in duplicate and SEM was estimated.

### CCA/tRNA^Phe^-catalyzed ribosomal peptide release assay

Unmodified full-length tRNA^PheB^ was prepared by transcription *in vitro* (24). Equal volumes in 30% acetone (v/v) of tRNA^Phe^/CCA and tripeptide RCs (0.152 μM) harboring a UAA stop codon for CCA release or a UUC codon for tRNA^Phe^ release in the A site were preincubated in Hepes–PM buffer at given pHs for 5 min at 37 °C. RNAs and RCs were mixed, and reactions were quenched at each given time point by 17% formic acid. Samples were processed as for RF1-catalyzed release (see aforementioned) except the time courses were fitted with a single exponential decay function

$$\text{y}=\text{A∗exp}\left(-\text{x/t}\right){+\text{y}}_{\text{0}}$$

where y is the release fraction, x is the time, A is the amplitude of the individual phases, t is the time constant, y_0_ is the offset, and 1/t is the rate constant (k). The pHs for titration experiments were 7.2 for CCA and 7.5 for tRNA^Phe^. The final concentrations of CCA and tRNA^Phe^ used for pH dependence experiments were 76 and 2 μM, respectively. All RNA titration data were fitted with the Michaelis–Menten equation. All experiments were conducted at least in duplicate, and SEM was estimated.

### Codon dependence of tRNA-catalyzed release in 30% acetone

Unmodified full-length tRNA^PheB^, full-length tRNA^AlaB^, and 3′CA-truncated tRNA^AlaB^ were prepared by transcription *in vitro* (24). RCs were prepared as described in the fMet-ester formation assay (see later) except for the GCA (Ala) A-site codon. Equal volumes in 30% acetone (v/v) at pH 7.5 of RCs (0.2 μM) and tRNAs (15.2 μM unless otherwise indicated in figure legends) were preincubated separately at 37 °C for 5 min. Release was measured by quenching with 17% of formic acid at given time points. Samples were analyzed as described in CCA/tRNA^Phe^-catalyzed ribosomal peptide release assay (see aforementioned). All experiments were conducted at least in duplicate, and SEM was estimated.

### RF1/CCA/tRNA^Phe^-catalyzed fMet-ethyl ester formation assay in 20% ethanol

RCs were prepared by mixing 2 μM 70S ribosomes, 5 μM mRNA, 2.4 μM f[^3^H]Met-tRNA^fMet^, 2 μM IF1, 2 μM IF2, and 2 μM IF3 at 37 °C for 20 min. The mRNA encoded either a UAA stop codon for RF1 or CCA-catalyzed esterification or a UUC Phe codon for tRNA^Phe^. Equal volumes (20 μl) in 20% ethanol (v/v) at pH 7.5 of RCs (0.2 μM) and RF1 (8 μM), CCA (152 μM), or tRNA^Phe^ (15.2 μM) were preincubated separately at 37 °C for 5 min. At the judged reaction end times (RF1: 10 s, CCA: 480 s, and tRNA^Phe^: 600 s), the samples were put on ice and divided into two aliquots of ∼20 μl: one portion was mixed with 200 μl 0.1 M imidazole, pH 6.0 (following the extraction method of Caskey *et al.* (10)), the other with 26 μl of 30% formic acid. Imidazole samples were extracted with 1.5 ml of ethyl acetate, centrifuged, and the organic phases were subjected to scintillation counting. Samples quenched with formic acid were analyzed as for RF1-catalyzed release (as aforementioned, but supernatants now contained both fMet and fMet-ethyl ester products; fMet yields were calculated by subtracting fMet-ethyl ester yields determined by the ethyl acetate extractions aforementioned). The pH-dependent rates with tRNA^Phe^ were measured from time courses. All experiments were conducted at least in duplicate, and SEM was estimated.

## Data availability

All primary data points come from Excel xls files automatically generated by a scintillation counter (Beckman Coulter LS 6500) in our laboratory. They are stored there, backed up on the computer of L. B. and printed out and pasted in the lab book of L. B. Primary data files are available upon request from L. B. and A. F.

## Supporting information

This article contains supporting information. Reference (20) is cited in the legend of Fig. S4.

## Conflict of interest

The authors declare that they have no conflicts of interest with the contents of this article.
