# Supplementary material for: Rate-limiting hydrolysis in ribosomal release reactions revealed by ester activation
Source: J Biol Chem. 2022 Sep 20;298(11):102509. doi: 10.1016/j.jbc.2022.102509 (PMC9589212; doi:10.1016/j.jbc.2022.102509)
Supplement: Supplemental Tables S1–S3 and Figures S1–S6 [file mmc1.docx]

SUPPORTING INFORMATION

**Rate-limiting hydrolysis in ribosomal release reactions revealed by ester activation**

Letian Bao, Victoriia V. Karpenko and Anthony C. Forster^1^

Department of Cell and Molecular Biology, Uppsala University, Husargatan 3,

Box 596, Uppsala 75124, Sweden.

^1^To whom correspondence may be addressed. E-mail: [a.forster@icm.uu.se](mailto:a.forster@icm.uu.se)

­

**CONTENTS**

**Figure S1.** RF1-catalyzed ribosomal peptide release at pH 7.5.

**Table S1.** Release rates catalyzed by RF1 at different pHs in Figure 2D for tripeptides containing MeAla and TFMeAla.

**Figure S2.** Acetone dependence of CCA-catalyzed ribosomal peptide release at pH 7.2.

**Figure S3.** Acetone dependence of tRNA^Phe^-catalyzed ribosomal peptide release at pH 7.5.

**Figure S4.** Dependence on codon and 3’CA of tRNA-catalyzed ribosomal peptide release.

**Figure S5.** CCA-catalyzed ribosomal peptide release at pH 7.5.

**Table S2.** Release rates catalyzed by CCA at different pHs in Figure 3D for tripeptides containing MeAla and TFMeAla.

**Figure S6.** tRNA^Phe^-catalyzed ribosomal peptide release at pH 7.5.

**Table S3.** Release rates catalyzed by tRNA^Phe^ at different pHs in Figure 4D for tripeptides containing MeAla and TFMeAla.


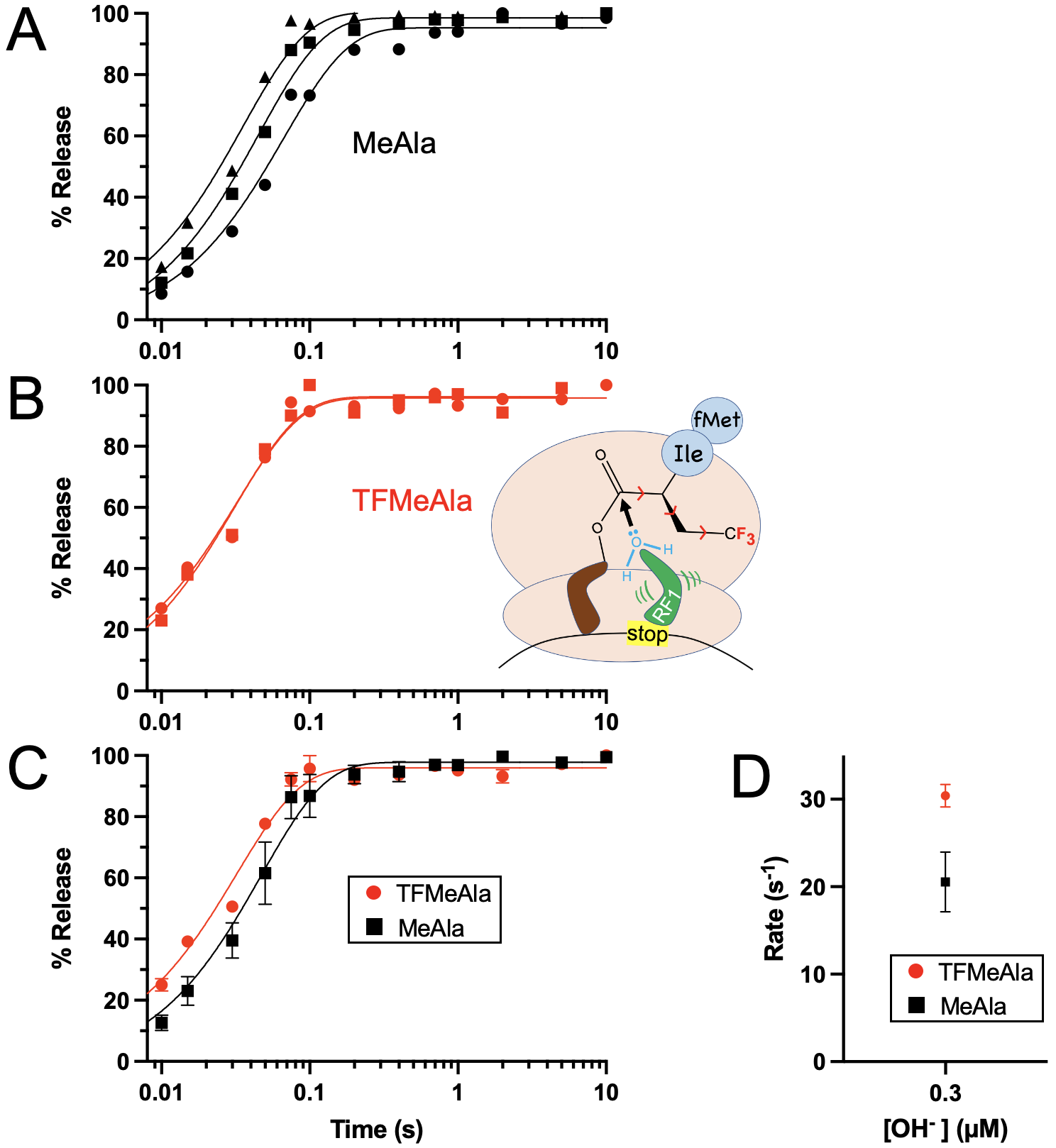


**Figure S1. RF1-catalyzed ribosomal peptide release at pH 7.5.** (A, B) Individual time courses of three and two independent experiments for MeAla (black) and TFMeAla (red), respectively. A cartoon of the activated RC in (B) is shown. (C) Combined time courses with weighted averages from A and B. (D) Rates calculated from C. Final concentrations of RCs = 0.1 μM and RF1 = 3 μM. Bars are SEs calculated from A and B. Spontaneous release rates (in absence of RF; not shown) were 0.012 ± 0.002 min^-1^ (MeAla) and 0.018 ± 0.003 min^-1^ (TFMeAla).


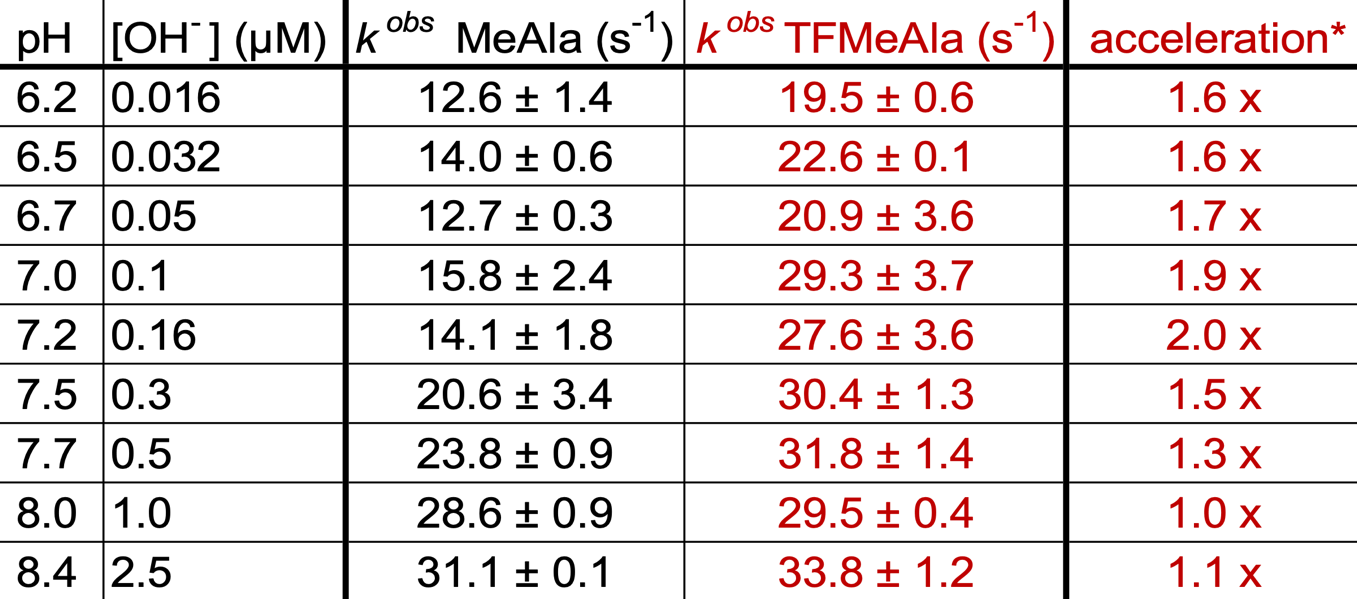


**Table S1.** **Release rates catalyzed by RF1 at different pHs in Figure 2D for tripeptides containing MeAla and TFMeAla.** The acceleration was calculated by dividing *k_TFMeAla_* by *k_MeAla_.* SEs were calculated based on at least two independent experiments.

* Theoretically, the observed times for reaction were a combination of times of two major steps, a conformational change and ester hydrolysis (τ^obs^ = τ_conf_ + τ_hydr_). Assuming the conformational change rate is independent of pH, constant and not accelerated, τ_conf_ = 1/33.8 s^-1^ = 30 ms, so τ_hydr_ = τ^obs^ – τ_conf_. At acidic pHs, TFMeAla accelerated τ_hydr_ overall ~2.5 x compared to MeAla, calculated as follows:

pH MeAla (ms) TFMeAla (ms) acceleration

6.2: 50.3 ± 9.0 / 21.2 ± 1.7 = 2.4 x

6.5: 41.6 ± 3.1 / 14.3 ± 0.1 = 2.9 x

6.7: 48.8 ± 1.9 / 19.5 ± 8.7 = 2.5 x

For pHs ≥7.0, however, TFMeAla rates nearly reached the conformational change plateau, making the subtracting calculation inaccurate.


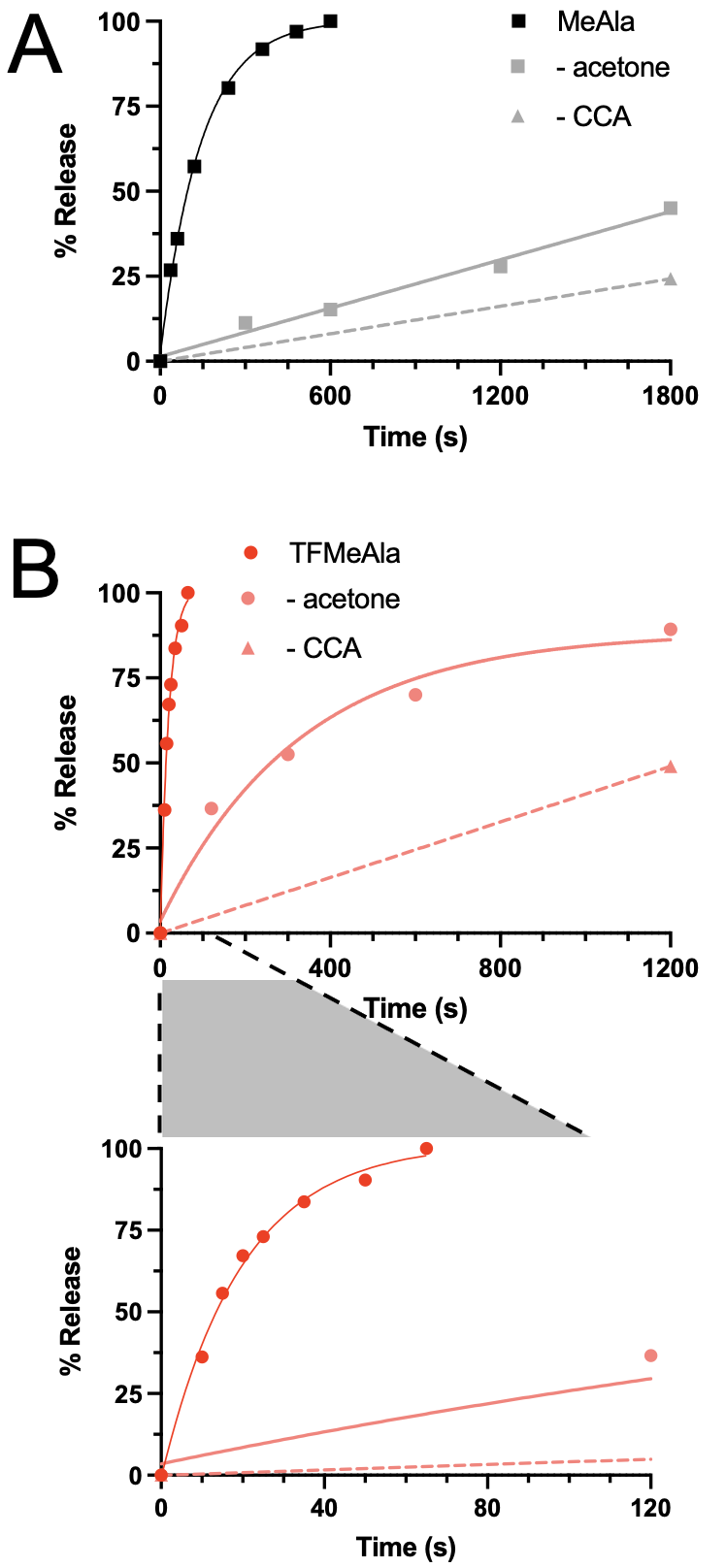


**Figure S2: Acetone dependence of CCA-catalyzed ribosomal tripeptide release at pH 7.2.** Representative time courses of (A) fMI-MeAla and (B) fMI-TFMeAla tripeptide released from RCs in the presence of 30% acetone compared with absence of acetone (fainter lines) or absence of CCA (dashes = spontaneous release). The bottom of B expands the first 120 s. Final concentrations of RCs = 76 nM and CCA = 76 μM.


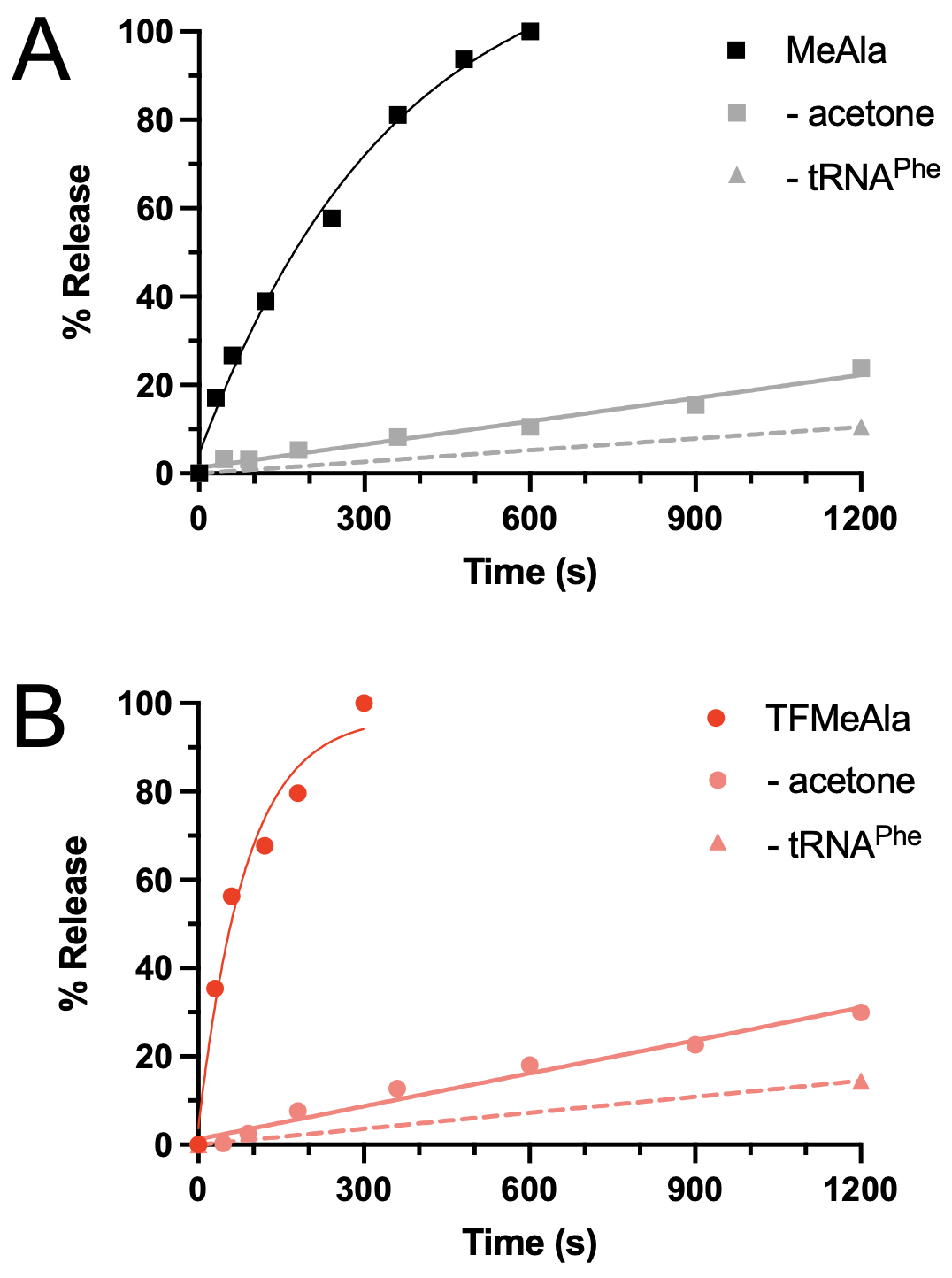


**Figure S3: Acetone dependence of tRNA^Phe^-catalyzed ribosomal tripeptide release at pH 7.5.** Representative time courses of (A) fMI-MeAla and (B) TFMeAla tripeptide released from RCs in the presence of 30% acetone compared with absence of acetone (fainter lines) or absence of tRNA (dashes = spontaneous release). Final concentrations of RCs = 76 nM and tRNA^Phe^ = 2 μM.


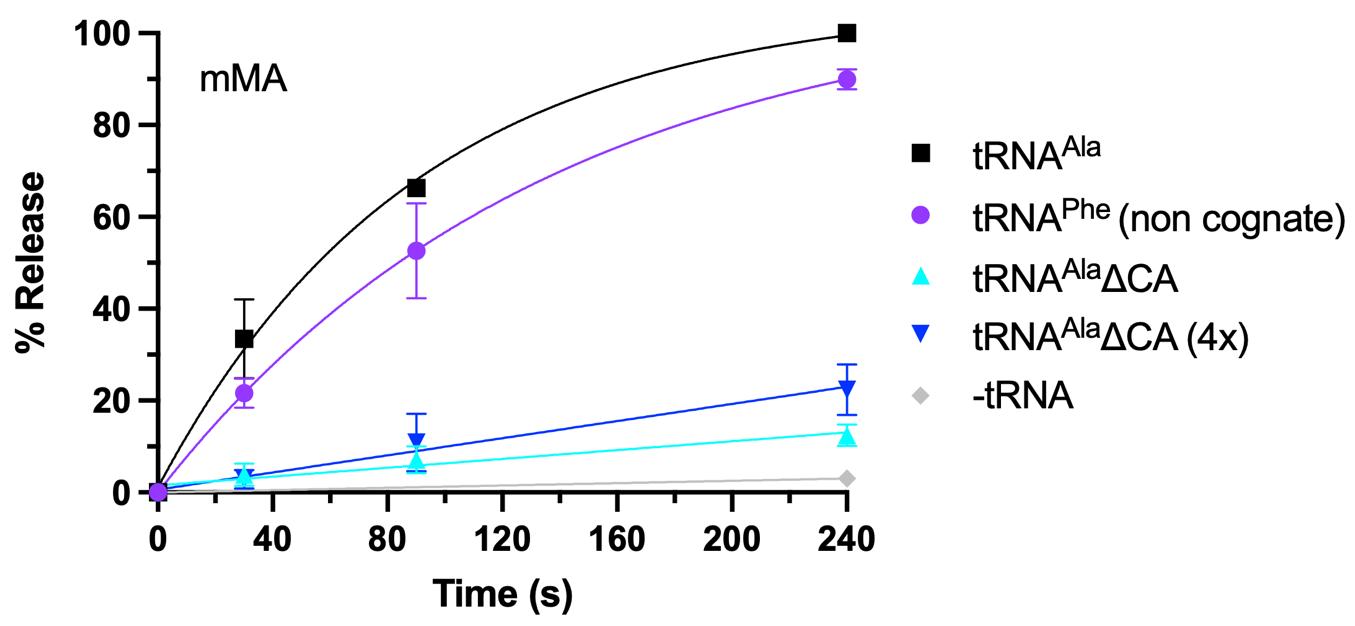


**Figure S4. Dependence on codon and 3’CA of tRNA-catalyzed ribosomal fMet release.** Release time courses of RCs (0.1 μM) harboring fMet-tRNA in the P site and mRNA MA with a GCA (Ala) codon in the A site in 30% acetone catalyzed by cognate tRNAAla UGC (7.6 μM), non-cognate tRNAPhe GAA (7.6 μM) or 3’CA-truncated cognate tRNAAla UGC (7.6 or 30.4 μM). Absence of tRNA = spontaneous release. SEs from at least two independent time courses. The release yields without acetone were low at 240 s (not plotted) and dependent on cognate tRNA^20^: 19% for cognate tRNAAla UGC, 3% for non-cognate tRNAPhe GAA and 2% for spontaneous.


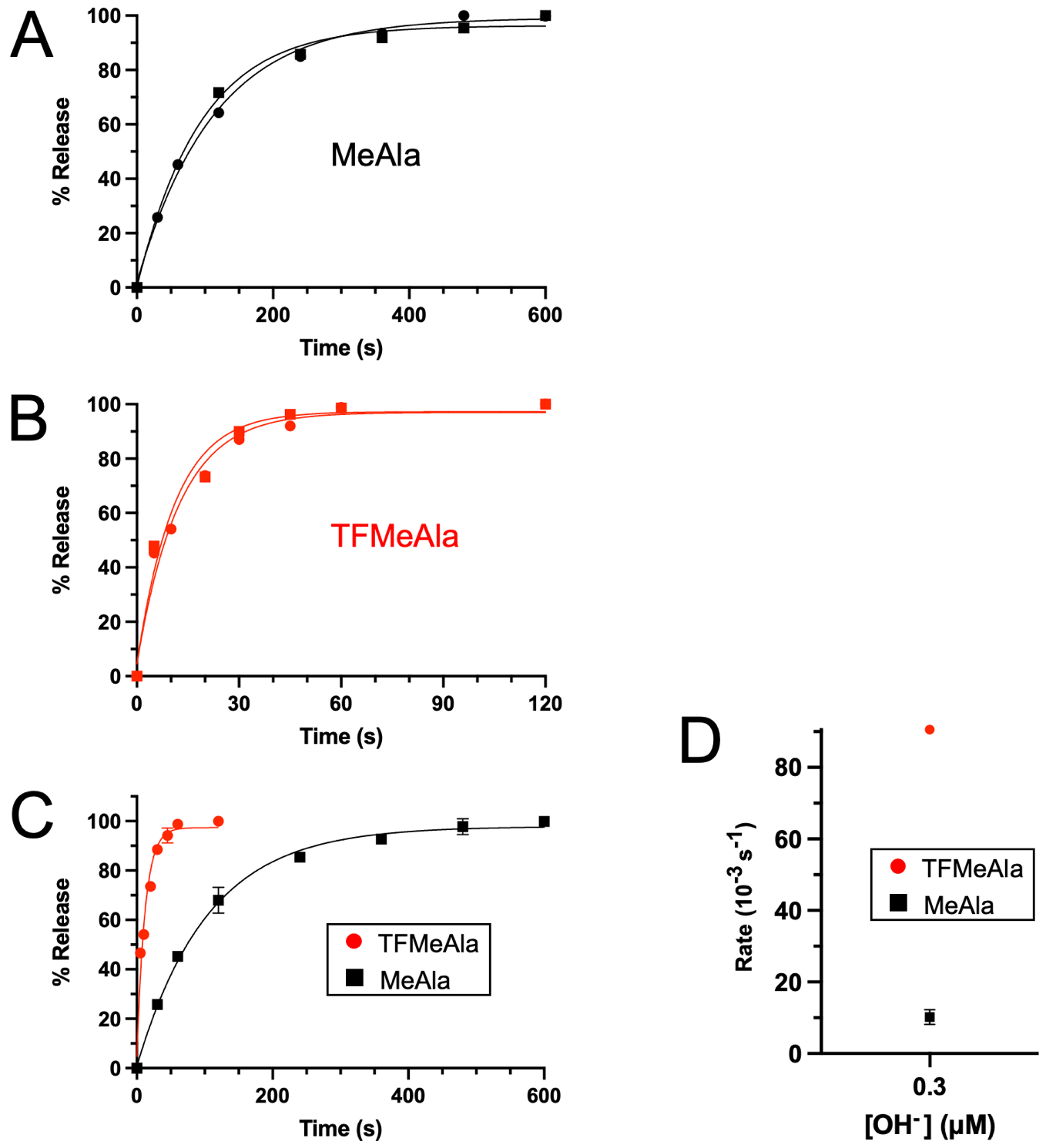


**Figure S5: CCA-catalyzed ribosomal tripeptide release at pH 7.5.** (A, B) Individual time courses of two independent experiments for MeAla and TFMeAla. (C) Combined time courses with weighted averages from A and B. (D) Rates calculated from C. Final concentrations of RCs = 76 nM) and CCA = 76 μM. Bars are SEs calculated from A and B.


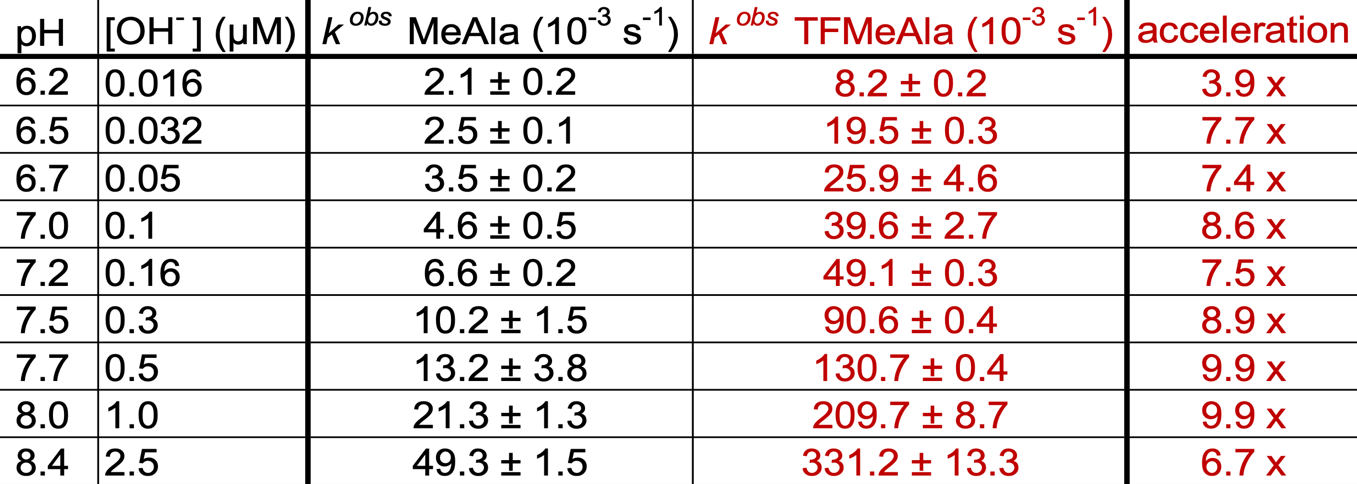


**Table S2.** **Release rates catalyzed by CCA at different pHs in Figure 3D for tripeptides containing MeAla and TFMeAla.** The acceleration was calculated by dividing *k_TFMeAla_* by *k_MeAla_.* SEs were calculated based on at least two independent experiments.

*
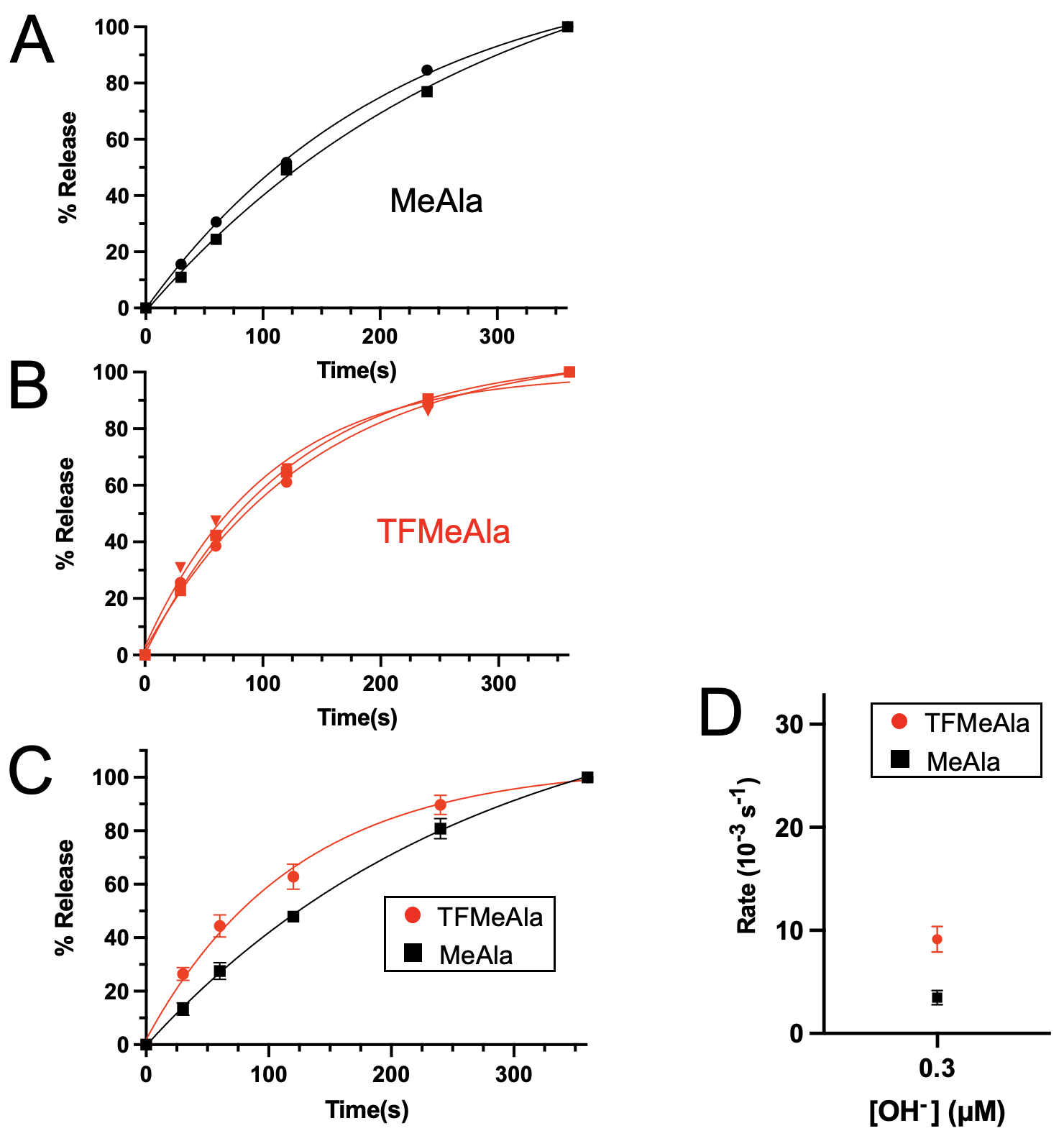
*

**Figure S6. tRNA^Phe^-catalyzed ribosomal tri peptide release at pH 7.5.** (A, B) Individual time courses of two and three independent experiments for MeAla and TFMeAla RC_UUC_s, respectively. (C) Combined time courses with weighted averages from A and B. (D) Rates calculated from C. Final concentrations of RCs = 76 nM and tRNA^Phe^ = 2 μM. Bars are SEs calculated from A and B.


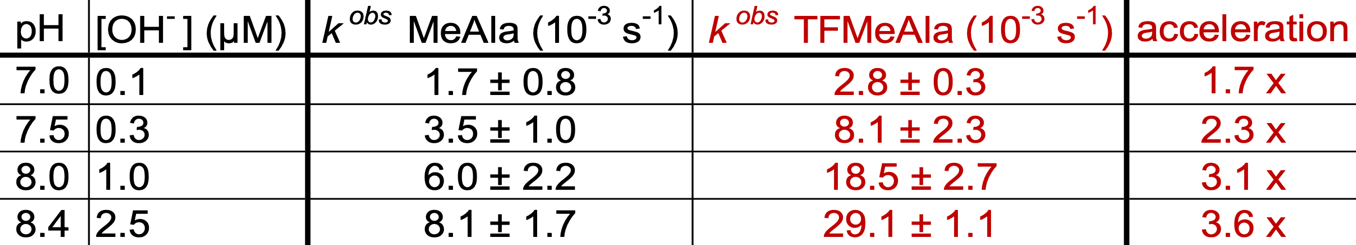


**Table S3.** **Release rates catalyzed by tRNA^Phe^ at different pHs in Figure 4D for tripeptides containing MeAla and TFMeAla.** The acceleration was calculated by dividing *k_TFMeAla_* by *k_MeAla_.* SEs were calculated based on at least two independent experiments.

----
